# Subjective impact of traumatic brain injury on long-term outcome at a minimum of 10 years after trauma– first results of a survey on 368 patients from a single academic trauma center in Germany

**DOI:** 10.1186/1754-9493-7-32

**Published:** 2013-10-10

**Authors:** Hagen Andruszkow, Julia Urner, Ezin Deniz, Christian Probst, Orna Grün, Ralf Lohse, Michael Frink, Frank Hildebrand, Christian Zeckey

**Affiliations:** 1 Department for Trauma and Reconstructive Surgery, University Hospital Aachen, Pauwelsstraße 30, Aachen 52074 Germany; 2 Department of Trauma and Orthopaedic Surgery, Cologne Merheim Medical Center, Faculty of Health-School of Medicine, Witten/Herdecke University, Ostmerheimer Straße 200, Cologne 51109, Germany; 3 Hannover Re Insurance, Karl-Wiechert-Allee 50, Hannover 30625, Germany; 4 Trauma Department, Hannover Medical School, Carl Neuberg-Str. 1, Hannover 30625, Germany; 5 Department for Trauma, Hand and Reconstructive Surgery, University Medical Center Marburg, Baldingerstr, Marburg 35043, Germany

**Keywords:** Traumatic brain injury, TBI, Outcome, SF-12, GOS

## Abstract

**Background:**

Traumatic Brain Injury (TBI) may lead to significant impairments in personal, social and professional life. However, knowledge of the influence on long-term outcome after TBI is sparse. We therefore aimed to investigate the subjective effects of TBI on long-term outcome at a minimum of 10 years after trauma in one of the largest study populations in Germany.

**Methods:**

The current investigation represents a retrospective cohort study at a level I trauma center including physical examination or standardized questionnaires of patients with mild, moderate or severe isolated TBI with a minimum follow-up of 10 years. We investigated the subjective physical, psychological and social outcome evaluating the Glasgow Outcome Scale, short-form 12, and social as well as vocational living circumstances.

**Results:**

368 patients aged 0 to 88 years were included. Patients with severe TBI were younger compared to patients with moderate or mild TBI (p < 0.05). Patients with severe TBI lived more often as single after the trauma impact. A significantly worse outcome was associated with higher severity of TBI resulting in an increased incidence of mental disability. A professional decline was analyzed in case of severe TBI resulting in significant loss of salary.

**Conclusions:**

The severity of TBI significantly influenced the subjective social and living conditions. Subjective mental and physical outcome as well as professional life depended on the severity of TBI 10 years after the injury.

## Introduction

Traumatic Brain Injury (TBI) is worldwide known as a major public health concern potentially resulting in death or neurological impairment [1,2]. The incidence of TBI is about 300 per 100,000 inhabitants [3] with almost 50% related to traffic accidents in the Western civilization [4].

Due to increasing clinical experience and improved treatment algorithms, overall mortality decreased during the last thirty years in traumatized patients [5]. Thus, research also focused on long-term outcome after major trauma including TBI. However, despite this increasing interest in research on long-term outcome following trauma in general, patients with TBI were frequently omitted from study populations due to the known impact on mortality [6]. Furthermore, many long-term outcome studies including patients with TBI exhibit potential limitations. First, research emphasizing on multiple trauma patients might not estimate the complexity of the impact of TBI as the presence of multiple injuries influences morbidity and long-term perceptions [5,7]. The same limitation might be observed in studies emphasizing TBI without excluding other severe injuries resulting in compromised comparisons between isolated and multiple traumatized patients of different injury severity [6,8]. In conclusion, only limited information on long term recovery and morbidity more than 10 years after isolated TBI are available [3,9-11]. Furthermore, these reports commonly focused only on the impact of mild TBI [3,9], moderate or severe TBI [10] or special subgroups [11] limiting general assumptions. Consequently, more comprehensive long-term outcome studies after isolated TBI are required in order to document potential prognosis and to prepare life plans for survivors, families and clinicians [10]. In the presented study we aimed to verify medical, social as well as vocational long-term outcome results after mild, moderate and severe TBI in one of the largest long-term outcome study populations after isolated TBI in Europe.

## Methods

The study was approved by the institutional ethical review board of the Hannover Medical School, Hannover, Germany, in 2010 (IRB No. 6221). Written informed consent was obtained from all adult participants. In case of children (aged <18 years), parental permission and child assent were used for participation. One or both parents accompanied the questioning and re-examination.

### Study design

The investigation was designed as a retrospective cohort study at a level I trauma center. The assessment and re-examination of the included patients was performed between December 1^st^ 2009 and October 31^st^ 2011.

### Participants

Patients were analyzed by our databank and included in the study if the following criteria were fulfilled: Only traumatized patients who have sustained TBI (GCS 3 - 15 points) were included. Minimum follow-up was at least 10 years after trauma. Patients aged 0 to 88 years were included.

In order to focus on the impact of isolated TBI we excluded patients who had sustained any additional injury defined by an Abbreviated Injury Scale (AIS) (version 2005) [12] larger 2. Because patients might have sustained additional injuries until follow-up due to the long time period we excluded patients who sustained any repeated trauma until follow-up with an AIS score larger than two points and any repeated TBI. Physical or mental handicap previous to TBI which was documented in the patients’ charts led to exclusion as well. Physical or mental handicap was defined as disability of loss of function including inability to communicate or to perform mobility, preventing from participation in any activities of daily living.

**Figure 1:**
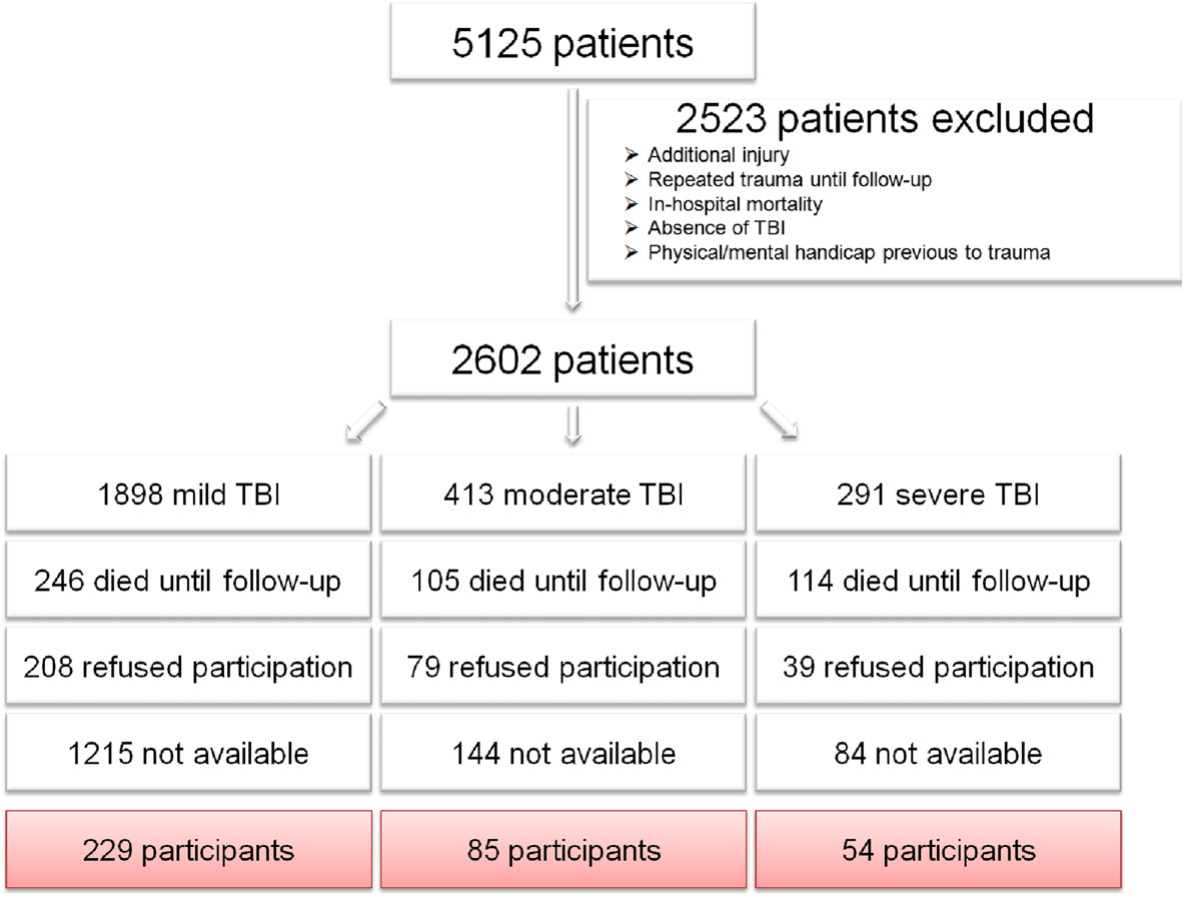
Flow chart.

The flow chart demonstrates the detailed inclusion process of this study (Figure 1).

#### Contacting of patients and examination

Patients were recruited according to an established recruitment process [13]: Apriori, patients’ residences were gathered from the charts. If patients had moved, up to three different registration offices were contacted by mail in order to determine the current address. Afterwards, the patients were contacted by mail in a letter describing the purposes of the present study and asked to make an appointment. The patients were contacted via mail and subsequently by phone up to three times. If none of these attempts was successful or three appointments were missed, patients were documented as “not available” to follow-up.

Patients with mild TBI were interviewed over the telephone while patients with moderate and severe TBI were re-examined by an experienced orthopaedic trauma surgeon. For re-examination a self-administered patient questionnaire and a standardized physical examination were used, which have been previously described [14]. The following measures and outcome parameters were raised by this study:

### Measures

#### Traumatic brain injury

TBI was classified based on the initial Glasgow Coma Scale (GCS) [15]. All patients were grouped into the three commonly used TBI severity groups: mild (GCS 13 – 15) with absence of a neurological deficit, moderate (GCS 9 – 12) and severe (GCS 3 – 8) [3,16,17].

#### Demographic data, injury severity and social living conditions

Demographic and clinical data were extracted from the patients’ charts including patients’ age and gender. In addition to the GCS classification of the TBI severity, we intended to provide further information of the injury severity. Therefore, we used the Injury Severity Score (ISS) and New Injury Severity Score (NISS) for patients with moderate and severe TBI [12,18]. Both scores represent the injury severity of TBI according to the AIS score and are highly associated with physical outcome and mortality [12,18]. As a diversification, the NISS has been evaluated to be more precise referring to the injury severity of TBI [12,18].

### Outcome parameters

#### Glasgow outcome scale

In order to assess the neurological outcome the *Glasgow outcome scale (GOS)* with the following description was used [19]:

Persistent vegetative state: Patient exhibits no obvious cortical function.

Severe Disability: (Conscious but disabled). Patient depends upon others for daily support due to mental or physical disability or both

Moderate Disability: (Disabled but independent). Patient is independent as far as daily life is concerned. The disabilities found include varying degrees of dysphasia, hemiparesis, or ataxia, as well as intellectual and memory deficits and personality changes.

Good Recovery: Resumption of normal activities even though there may be minor neurological or psychological deficits.

#### Short form 12 (SF-12)

Furthermore, the post-trauma quality of life was observed. Therefore, the short form 12 (SF-12) was used for patient assessment as a modified version of the SF-36 in German language [20]. It implies Physical Component Summary Scale (PCS) and Mental Component Summary Scale (MCS) [21]. For children, school work was interpreted as professional work because daily school work can be interpreted safely as daily mandatory activation.

#### Social living condition

Regarding the social living conditions*, marital status* as well as *housing situation* were analyzed during follow-up by the previously mentioned questionnaire. Housing situation focused on rent accommodation and potential confinement in bed as well as the subjective estimate to perform common housework. The item *“fewer friends”* intends to verify if a subjectively reported decline of social contacts with close persons that the patient perceives as friendships could be analyzed [5]. All social aspects are presenting subjective associations towards the impact of TBI.

#### Vocational living condition

*Professional decline* refers to a subjective estimation of professional descent due to trauma sequelae either with or without professional retraining. The incidence of *professional retraining* was noted additionally.

*Unemployed patients* were defined as patients who were not able to work in their pretrauma job because of injury sequelae or were dismissed because of sick leave times when they were generally able to work.

*Salary decrease* intends to verify if a significant loss of regular salary could be evaluated due to the impact of TBI.

In general, children were excluded from the vocational analysis.

All vocational aspects are presenting subjective associations towards the impact of TBI.

#### Statistical analysis

The data were analyzed using the Statistical Package for the Social Sciences (SPSS; version 20; IBM Inc., Somers, NY, USA). Incidences are presented with counts or percentages while continuous values are presented as mean ± standard deviation (SD). Differences between the groups were evaluated with analysis of variance (ANOVA) for continuous data, while Pearson’s *χ*^2^-test was used for categorical values. A two sided p-value < 0.05 was considered to be significant.

## Results

### Patients’ recruitment

The detailed flow chart of patients’ recruitment is illustrated in Figure 1: Overall 2,602 patients were analyzed to be potential candidates to participate in the study. 465 patients (17.9%) died before follow-up visit. In addition, 1,443 patients (55.5%) were not available or did not react to the invitations due to unknown reasons. 326 patients (12.5%) refused to participate the study. Finally, a total of 368 patients (14.1%) was successfully enrolled for this study and completely re-examined either by telephone or by physical examination.

### Demographic data and social living conditions

Of the included 368 patients, 229 patients were classified with mild TBI, 85 patients with moderate TBI and 54 patients with severe TBI. Those patients with severe TBI were significantly younger and more often of male gender compared to patients with moderate or mild TBI (Table 1). Patients suffering from severe TBI demonstrated an increased ISS and NISS.

**Table 1:** Demographic and social data of 368 individuals after TBI

	**Mild TBI**	**Moderate TBI**	**Severe TBI**	**p-value**
**Number of patients**	**229**	**85**	**54**	**-**
Age at time of injury (years)				
Mean ± standard deviation	23.7 ± 16.6	29.0 ± 20.9	22.5 ± 16.4	0.038
Range (minimum - maximum)	1.0 - 66.0	1.0 - 76.0	1.0 - 60.0	
median	20.0	28.0	19.0	
Age at time of reexamination (years)				
Mean ± standard deviation	44.2 ± 16.9	43.4 ± 20.08	26.5 ± 16.5	0.018
Range (minimum - maximum)	12.0 - 87.0	11.0 - 87.0	12.0 - 76.0	
median	41.0	42.0	34.5	
Time to reexamination (years)				
Mean ± standard deviation	20.3 ± 6.5	14.2 ± 4.1	14.0 ± 3.9	<0.001
Number of patients <18 years	101	33	26	0.531
(%)	(44.1%)	(38.8%)	(48.1%)	
Gender distribution (♂: ♀)	129: 100	57: 28	40: 14	0.026
Injury Severity (ISS)	-	14.8 ± 7.5	21.4 ± 7.1	<0.001
New Injury Severity (NISS)	-	24.3 ± 12.3	36.3 ± 14.8	<0.001
Marital status at follow-up				
- Single	40.7%	42.2%	64.8%	0.010
- Married or cohabitant	51.0%	50.6%	24.1%	
- Divorced	8.3%	7.2%	11.1%	
Housing situation before TBI				
- in rented accommodation	51.2%	46.8%	38.5%	0.249
Housing situation at follow-up				
- in rented accommodation	50.2%	42.0%	38.5%	0.205
- confinement in bed	0.0%	0.0%	5.6%	<0.001

♂Male.

♀Female.

Evaluating the living conditions, patients with severe TBI lived significantly more often as singles and divorced individuals after trauma impact (Table 1). With regard to the circle of friends, severe TBI led to a significant loss of friendships after trauma (mild TBI: 9.2%, moderate TBI: 11.3%, severe TBI: 31.4%; p < 0.001).

According to the housing situation, no differences referring to the rent accommodation was elucidated between the TBI groups. However, continuous confinement in bed was strongly associated to severe TBI (Table 1).

### Outcome measurement

According to the GOS, a significantly worse outcome was associated with higher severity of TBI (Table 2). Furthermore, an increasing incidence of mental disability was evaluated in the presence of moderate or severe TBI. No differences were elucidated according to the SF-12, neither for Physical Component Summary Scale nor Mental Component Summary Scale between the different severity of TBI.

**Table 2:** Outcome measurement according to the injury severity of TBI

	**Mild TBI**	**Moderate TBI**	**Severe TBI**	**p-value**
GOS (points)	5.0 ± 0.0	4.9 ± 0.4	4.3 ± 0.8	<0.001
Mental disability	0.0%	1.2%	5.6%	0.004
SF-12 (PCS)	43.8 ± 5.3	42.5 ± 5.2	42.9 ± 6.3	0.181
SF-12 (MCS)	55.0 ± 10.8	54.3 ± 9.1	52.2 ± 8.9	0.243

### Vocational living condition

A significant professional decline was analyzed in case of severe TBI with almost half of the patients describing an occupational descent due to trauma sequelae (Table 3). Relating to the worsening of the vocational situation, significant loss of regular salary was associated with higher TBI severity. However, a significant increase of professional retraining as well as unemployment could not be found between the TBI groups.

**Table 3:** Vocational situation according to the injury severity of TBI

	**Mild TBI**	**Moderate TBI**	**Severe TBI**	**p-value**
Occupational decline	11.7%	15.3%	41.0%	<0.001
Salary decrease	11.9%	17.4%	30.6%	0.025
Professional retraining	4.7%	4.9%	5.4%	0.984
Unemployment	6.5%	3.6%	12.9%	0.244

## Discussion

The current paper presents first results of one of the largest long-term outcome studies after isolated TBI in Europe. With the intention to verify medical, social as well as vocational long-term deficits following TBI in survivors our results can be summarized as follows:

Patients with severe TBI were significantly younger and more often of male gender than those with moderate or mild TBI. The analysis of living conditions revealed more individuals living alone in the severe TBI population after trauma compared to the other TBI groups. A significantly worse outcome according to the GOS as well as a higher incidence of mental disabilities was found after severe TBI. Patients with severe TBI were more often confined to bed than patients after moderate or mild TBI. Severe TBI significantly impacts vocational situation due to an occupational decline resulting in loss of regular income. Moderate and severe TBI were not associated with increased unemployment or professional retraining compared to patients with mild TBI.

Outcome after TBI has been investigated in different settings [3,9-11,16,17]. However, knowledge of the influence of isolated TBI on long-term outcome remains sparse due to several reasons. First, the studies varied considerably according to the definition of “long-term” with a posttraumatic observation period between 5 and 15 years [9,16,17]. Furthermore, some studies focused either only on the impact of mild TBI [3,9] or on the combination of moderate and severe TBI [10,16], whereas others verified outcome results only in multiple traumatized patients [5,7]. Third, due to the high mortality after severe TBI even 10 years after trauma [1] and the reduced probability of severe isolated head injuries after high energy trauma [4,22], included study populations used to be relatively small.

In the presented study we were obviously faced with the same problem: Almost 50% of the overall study population had to be excluded initially mainly because of in-hospital mortality or the presence of major concomitant injuries. The inclusion rate of 14.1% in our study is in line with current literature. For instance, Cameron et al. analyzed the 10-year outcome after TBI excluding not explicitly further injuries [6]. Identifying an overall potential population of 21,032 patients based upon a Canadian state registry, finally 1,290 took part in the re-examination (6.1%) [6]. In addition, Andersson and colleagues analyzed 198 patients evaluated from a main population of 1,719 patients with mild TBI (11.5%) [9].

According to the demonstrated demographic results, patients suffering severe TBI were significantly younger and more often of male gender compared to those with moderate or mild TBI. This over-representation of young and male trauma victims has been elucidated in isolated TBI as well as in multiple trauma patients previously [6,8,10]. One explanation for the increased incidence of severe injuries in these patients might be argued by the relatively high frequency of traffic accidents [4] as especially young male patients are known to be involved in high energy trauma [4]. In this context, road traffic accidents have been found responsible for up to 80% of TBI patients [4].

The living situation after isolated TBI is suspected to be a critical factor for quality of life and daily living activities. Living alone may be a sign of social isolation, but it may also reflect independence [10,16]. Nevertheless, it seems unlikely to expect that patients surviving severe TBI would be more capable of independent living than those with minor head injuries [10]. In this context, we found more “singles” and divorced individuals after severe TBI compared to moderate or mild TBI. Therefore, the prevalence of living alone presumably reflects social isolation. This suggestion has been also considered by Colantonio et al., who found individuals living alone in up to 60% after moderate to severe TBI [10]. In addition, even after isolated mild TBI an increased incidence of separated relationships has been found: Moreover, the measured incidence of 7% divorced patients meets the presented results (8.3%) [9]. However, as children were also included in the present study, many participants could still be living with their parents. This may be an indicator of more supportive environment but also could indicate compromised independence [8,10]. The latter aspect is strongly supported by the measured incidence of patients who were confined to bed due to TBI sequelae and those stating an inability to manage common homework in the presented study.

Focusing on long-term outcome measurements, the presented data indicates that survivors of severe TBI had significant impairments: These patients achieved significantly reduced GOS-scores accompanied with increased incidence of mental disability. These findings are congruent with the current literature. Colantonio et al. compared mental tests of patients after moderate to severe TBI with normative expected results revealing increased mental disabilities after moderate and severe TBI [10]. In addition, Jacobsson et al. demonstrated reduced quality of life and impairment after moderate to severe TBI compared with a normative reference sample [16]. Interestingly, in the present study we did not find a significant difference in the quality of life measured by SF-12 between the different TBI groups despite the reported increased impairments measured by GOS. This is in contrast to other studies and might be based on the fact, that we aimed to compare the three severities of TBI rather than matching one of them to a normalized population as other studies did [10,16]. In this context, a profound influence or diminished quality of life due to mild TBI 10 years after injury is debatable in the current literature: Accordingly, Sadowski-Cron et al. revealed persisting complaints such as headache, concentration deficits and somatic complaints [8]. Furthermore, Zumstein et al. were able to verify mild TBI impacting life quality 10 years after trauma considerably due to posttraumatic somatic syndromes [3]. These complaints have been demonstrated to result in reduced SF-12 scores compared to normative population [9].

Unemployment is known as a significant problem following TBI [3,10]. According to the presented results, severe TBI resulted in a significant occupational decline followed by loss of salary. Interestingly, the demonstrated comparable unemployment status between the different TBI groups has not been found in the current literature: Grauwmeijer et al. revealed that patients after moderate and severe TBI with impaired cognitive functioning at hospital discharge were at high risk of long-term unemployment three years later [23]. These findings were supported by the long-term follow-up study of Jacobsson et al., whose patients with moderate to severe TBI had increasing unemployment rates compared to mild TBI [16]. However, these studies evaluated unemployment rates up to 44% after severe TBI which seems considerably higher compared to the presented rate of approximately 13%. Comparability to the presented results could be limited due to increased disability rates measured by GOS: The authors found up to 80% of patients with severe TBI had a GOS less than 4 points meaning that 80% were severely disabled [16,23] while permanent disability in the presented study was revealed only in 5.6%. Furthermore, it might be assumed that significant reasons for these diverse results are found in the different health care and social systems [24], which make an international comparison and a prediction of the long-term vocational impact of different TBI severities difficult.

The presented study has several limitations. Due to the follow-up period of at least 10 years, many critical events might have occurred in a persons’ life potentially affecting outcome. Although the participating patients have been asked for life-changing events between the TBI and follow-up, this aspect has to be considered as a potential limitation when interpreting the results. Especially pre-existing psychological and behavioural problems might be missed by this study, because none of the traumatized patients was assessed by specific psychological scores on admission when treated for TBI. We excluded patients with mental handicaps previous to TBI, but minor psychological problems were potentially missed by this study. As these problems might interfere with the presented outcome results, this aspect should be taken into account when interpreting the presented results.

Another major limitation is to be mentioned by including pediatric trauma patients to the study population. Pediatric TBI is known to have a better physical outcome compared to adult patients due to the plasticity of the immature brain [11,17,25-31]. Although this aspect is not proven in the literature without remaining criticism [11,17,25-31], this study could have been biased considerably. However, we demonstrated the contingent of children between the TBI groups as statistical comparable reducing the bias effect. Nevertheless, results should be interpreted carefully due to this limiting factor.

Furthermore, the length of follow-up and data collection at a single center and its retrospective design might be a limitation and it is likely that the presented findings cannot reflect the advances made in acute care as well as rehabilitation during the last decades. Additionally, one might be aware of a potential selection bias due to the large number of excluded patients which is a known limiting aspect of long-term outcome studies discussed previously.

## Conclusion

According to the presented results, patients after severe TBI are confronted with social living, vocational and outcome restrictions. Knowledge of these impairments might regulate further life plans of TBI relatives in order to create more supportive living environments minimizing social isolation. Furthermore, occupational rehabilitation or financial insurance support might be aspects that could limit the financial burden following the occupational decline after severe TBI. In addition, due to the revealed disability after severe TBI the considerable status of physical and mental rehabilitation could be expected emphasizing on the need of social and vocational reintegration.

## Competing interests

Industrial support was provided by Hannover Life Re-Insurance, Hannover, Germany. Dr. Nicola-Alexander Sittaro, MD, and Dr. Ralf Lohse, PhD, Hannover Life Re-Insurance, Hannover, Germany, gave advice. No direct or indirect financial support or other assets were transferred to the authors for this study. The authors state that there are no competing interests.

## Authors’ contributions

HA conceived this study designing the trial, provided statistical advice on study design, analyzed the data and drafted the manuscript. CP, OG, RL and MF provided statistical advice on the study design, analyzed the data and supervised the conduct of the trial and data collection. JU and ED conceived the study and designed the trial. CZ conceived the study, designed the trial, obtained research funding and supervised the conduct of the trial. FH conceived the study, designed the trial, obtained research funding, supervised the conduct of the trial and data collection, provided statistical advice on study design and analyzed the data. HA takes responsibility for the article as a whole. All authors contributed substantially to manuscript revision. All authors have read and approved the final manuscript for publication.
